# Indoor spectroradiometric characterization of plastic litters commonly polluting the Mediterranean Sea: toward the application of multispectral imagery

**DOI:** 10.1038/s41598-020-74543-6

**Published:** 2020-11-16

**Authors:** L. Corbari, A. Maltese, F. Capodici, M. C. Mangano, G. Sarà, G. Ciraolo

**Affiliations:** 1 Dipartimento di Scienze della Terra e del Mare, Università degli Studi di Palermo, Palermo, Italy; 2 Dipartimento di Ingegneria, Università degli Studi di Palermo, Palermo, Italy; 3 Stazione Zoologica Anton Dohrn, Dipartimento di Ecologia Marina Integrata, Sede Interdipartimentale della Sicilia, Lungomare Cristoforo Colombo (Complesso Roosevelt), 90142 Palermo, Italy

**Keywords:** Environmental impact, Optical spectroscopy

## Abstract

Around 350 million tonnes of plastics are annually produced worldwide. A remarkable percentage of these products is dispersed in the environment, finally reaching and dispersed in the marine environment. Recent field surveys detected microplastics’ concentrations in the Mediterranean Sea. The most commonly polymers found were polyethylene, polypropylene and viscose, ethylene vinyl acetate and polystyrene. In general, the in-situ monitoring of microplastic pollution is difficult and time consuming. The main goals of this work were to spectrally characterize the most commonly polymers and to quantify their spectral separability that may allow to determine optimal band combinations for imaging techniques monitoring. The spectral signatures of microplastics have been analysed in laboratory, both in dry condition and on water surface, using a full spectrum spectroradiometer. The theoretical use of operational satellite images for remote sensing monitoring was investigated by quantifying the spectral separability achievable by their sensors. The WorldView-3 sensor appears the most suitable for the monitoring but better average spectral separability are expected using the recently released PRISMA images. This research was preparatory to further outdoor experiments needed to better simulate the real acquisition condition.

## Introduction

The production and consumption of plastics in the world is growing in the last decades reaching 348 million tonnes in 2017^1^. Because of the high request of plastic material, the production is expected to be the quadruple of 300 million tonnes in 2050^2^. The higher plastic demand is meet by industrial sector producing short lifetime packaging^1^. In fact, 40% of total plastic production is represented by single-use packaging^2^. Moreover, packaging becomes a waste in about a year from its production^3^. One of the most important problems linked with the plastic production is the dispersion at sea. In fact, the solid waste discharging in rivers, costal zones or marine environment cause training of marine litter^4^. Although Europe is involved in the waste management with ever strict rules, a portion of plastic waste still ends up in the sea^5^. Indeed, the marine litter is composed from 50 to 90% by plastics^6^ and it is known that more than ten million tonnes of plastic reach the ocean each year^5^. The dispersion of plastic materials in the environment is leading to an increasing problem around the world. The sea surface and the deep sea currents cause a dispersion and more than one hotspot of plastics on the sea^5^. Specifically, in a semi-enclosed sea such as the Mediterranean Sea there is an accumulation of floating litter^7^.

The composition of the marine litter is variegated and it is known that microplastics on the sea are more than 92% of all plastic items^8^. As a function of the origin, microplastics can be classified in: (1) primary, which are directly introduced in the sea as particles smaller than 5 mm and (2) secondary, which are generated by the degradation of bigger plastics litter within the marine environmental^9^. An example of source of primary microplastics is represented by particles inside the cosmetics^10^ such as toothpaste, cosmetics, synthetic textiles^11^ whose small size facilitates their conveyance through wastewaters. At the end, however, every plastic particle—regardless the size and nature—converges towards the sea habitats^12^ making this material ubiquitous around the globe and omnipresent in rivers, estuaries and shores. Once at sea, microplastics are dispersed through sea currents, temporarily accumulating in the ocean surface and later along the water column and, finally, reaching the seabed^5^. Along its path and/or once reached their final destination (e.g. seafloor^13^) with differential times depending on size, density, abundance, shape, colour and the ultimate bio-availability^14^, plastics canalise along food webs affecting water column and seabed integrity, ecological equilibriums, as whichever source of pollution. The trophic transferring begins always through direct effects on the functional (individual) level—by affecting ingestion, growth, fecundity and mortality rates of marine organisms^15^—thereby propagating along the ecological hierarchy^16^ affecting community and ecosystem levels, and finally impairing the ecosystem functioning. Biodiversity is highly impaired by plastics as showed by more than 1400 marine species known to interact with marine plastic litter in different ways^17^. As a main consequence, through the effect on biodiversity, plastics can affect the relationship between biodiversity and ecosystem functioning which impairs the provision of ecosystem good and services and, at the end, the human well-being^18,19^.

Nonetheless, even though there is a growing overall concern around these plastic issues, yet there is not a standardised method to recognise plastic materials at sea at large scale than that of microscale (i.e. local; less than some dozens of meters) and the detection is usually performed through visual observation from boats or ships^20^. The ability to detect plastics (also microplastics) at sea surface is beneficial as it could inform and drive proactive mitigation management plans around the world by increasing the early detection accuracy of plastic hotspots, “sources” areas, once over the marine surface. Thus, it is becoming always more pressing to study the spatial and temporal distribution of plastic litter to start measuring the effects on coastal social, economic, and environmental systems. Indeed, the early detection and the near real time mapping of floating litter may represent a first informative layer that, once integrated on marine spatial planning framework, increase the stakeholders’, environmental practitioners’ and decision makers’ ability to mitigate effects on protected/vulnerable habitat and species^21,22^. Such a layer may assist in reducing the detrimental potential on societies and economies relying on marine ecosystem services (e.g. fisheries, aquaculture and tourism^23,24^).

To improve the remote sensing technology, by increasing the ability of analysis to estimate the plastic concentrations at sea surface, could facilitate the detection of seafloor accumulation hot-spots^5,25^. One solution to remedy the technological limitation comes from shortwave infrared imagery acquired by an airborne to detect plastics litter at the sea. Such a technological option could make possible the discrimination of plastics from the seawater, although to improve it and to provide a generalised information, there is the need to know the spectral signature of several polymers^26^. The plastics litter release the surfactants in the sea during their degradation. These change the fluid-dynamic propriety of the sea that could be detected from the COSMO-SkyMed SAR image^27^ but, before to effectively use remote sensing technology for monitoring, it is necessary to perform further investigation. Currently, it is possible to identify with satellite image the large plastics litter but is difficult to identify smaller plastics (debris) such as microplastics or nanoplastics.

Recently, the use of hyperspectral imaging (HSI) to detect and identify plastics debris was explored^28,29^. In particular, some authors highlighted that wavelengths, λ (nm), from 1000 to 2500 nm are suitable to recognise dry plastics above 300 µm by means of principal component analysis^29^; other authors applied a Partial Least Squares Discriminant Analysis techniques^28^ to HSI images to classify plastic samples.

In this framework, our goal was to identify the more suitable spectral bands to distinguish the different types of plastic polymers in order to give indications to authors focused on this hot-topic. To this aim, laboratory spectral experiments were carried out on plastic polymers frequently identified in the waters of the Mediterranean Sea. Indeed, a recent study of the Italian Research Council^30^ reporting on data collected through oceanographic cruises carried out in 2016 and 2017, revealed that plastic litters in the Mediterranean Sea belonged to 14 different polymers: polyethylene (PET) represented about 52%, polypropylene (PP) and viscose accounted for 17% and EVA-ethylene vinyl acetate and polystyrene (PS) for 6% and 3%, respectively. Further, about 67% of the samples was characterized by a dimension between 1 and 5 mm, 21% was smaller than 1 mm and 12% was bigger than 5 mm. The high presence of these and others polymers in microplastics and meso-plastics (5–20 mm) size in the Mediterranean sea is confirmed by other experimental ad hoc surveys^2^. Thus, accordingly, we designed an experiment to test the spectral separability of previously listed polymers, plus high-density polyethylene (HDPE), both in dry conditions and with plastics floating in water. The main aim was to select polymer’s bands in order to verify whether it will be possible to detect the presence of microplastics in the seawater through remote sensing imagery. To test it, we also compared present experimental spectral separability data dealing with each selected polymer with that used by the last generation of remote multispectral and hyperspectral sensors. The information gathered in the present study will be crucial to increase our understating about whether remote sensing can be useful to serve as an early detection tool when assessing the presence of microplastic litters at sea to inform the decision makers for designing management plans from local to regional scale.

## Results

This section reports results from the dry microplastics and microplastics on water experiments. Then, the feasibility of detecting plastic debris with current satellite sensors is discussed.

### Dry microplastics

The spectral signatures of microplastics samples were firstly evaluated setting an indoor spectroradiometric experiment.

**Figure 1:**
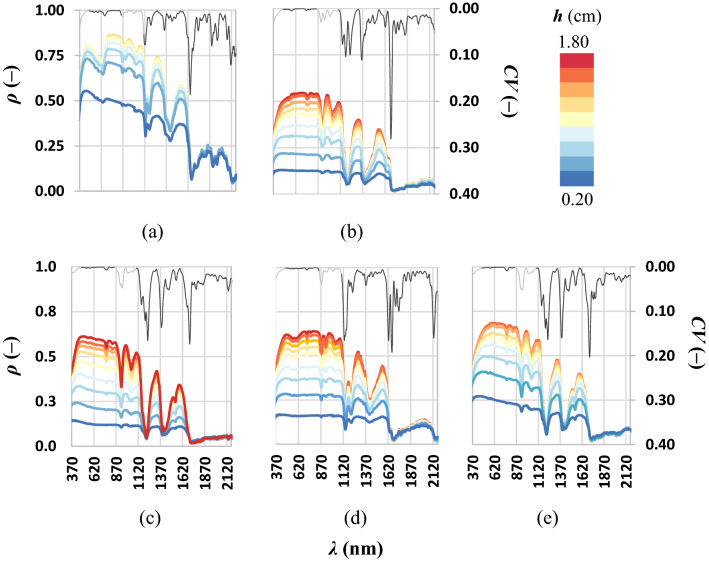
Spectral signatures of microplastics: (**a**) PET, (**b**) PP, (**c**) EVA, (**d**) PS and (**e**) HDPE, for increasing layers depth *h*. Light grey lines represent the *CV* averaged at 20 nm, while the *CV* values in selected spectral windows are represented in dark grey.

The total thickness *h* (cm) of plastic layers was equal to 1.2, 1.6 cm for PET and HDPE, respectively; whereas, for EVA, PS and PP the ring was fulfilled to minimize the effect of the underneath (black) panel on the emerging radiance. Spectral signatures of polymers with changing total thickness (color scale) with over-imposed average of the coefficient of variation *CV* (–) (grey line) are shown in Fig. 1.

Indoor spectral measures were almost noiseless as quantified by low *CV* occurring in two selected spectral windows: 480–850 nm and 1100 and 2170 nm (≈ 0.002–0.003 and ≈ 0.035–0.043, respectively); high *CV* values are due to steep variability of ρ (–).

The black panel underneath the plastic layers (figure not shown), used to minimize the influence of the support on the polymers reflectance, had an average reflectance ρ (–) of 4.5%, while spectrally, its reflectance was on average ≈ 0.03% from 350 to 640 nm. It had a small jump (≈ 0.008%) from 640 to 800 nm and then it increased linearly between 800 to 2100 nm (≈ 0.008%); finally, in the range from 2100 to 2500 nm the signal was very noisy.

**Figure 2:**
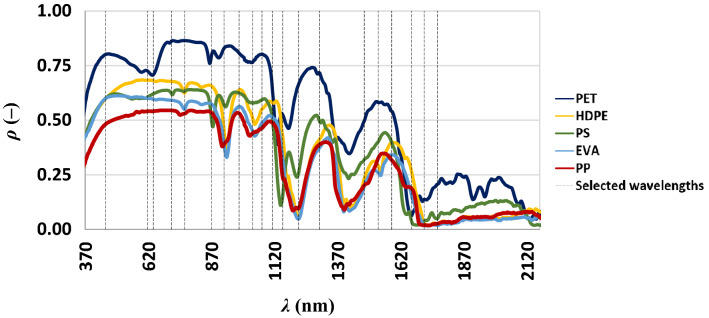
Spectral reflectance of dry microplastic at plateau.

For all microplastics, as the layers total thickness increases, ρ (–) increases as well (Fig. 1). The percentage increasing of ρ (–) per unit of additional layer progressively reduces. Further, the highest spectral signature was the sole used for the separability analysis. The reflectance of all polymers was higher in the VIS-NIR windows and lower in SWIR. Two spikes to minima of reflectance at 1400 and 1700 nm were evident for all polymers suggesting that these wavelengths could be not useful for outdoor passive detection. These reflectance minima corresponded to bands of adsorption as observed by Balsi et al.^31^. These spikes could be caused by the primary materials with which the plastics materials were made. Due to noises in measures below ~ 370 nm and above ~ 2100 nm, spectral reflectances of all materials were shown in a reduced spectral range. Spectral reflectances of all materials at maximum total thickness were compared to select a limited number of wavelengths (21) for the separability analysis (black vertical bars, Fig. 2).

Each polymer showed to have an initial low reflectance at 370 nm, then it increased linearly reaching a plateau; in the other parts of the VIS range the reflectance was almost constant. Accordingly, the opacity and the color of the polymers were different in VIS region:

- The PET exhibited major reflectances, indeed in the visible it was an opaque white;
- The PP had lower reflectance values because of it was greyer (in the visible) than the other polymers;
- The PS and EVA had similar reflectances as they were semi-transparent;
- The spectral signature of the HDPE was similar to the spectral signature of the PET but the values of HDPE were lower because it was less bright than PET.

**Figure 3:**
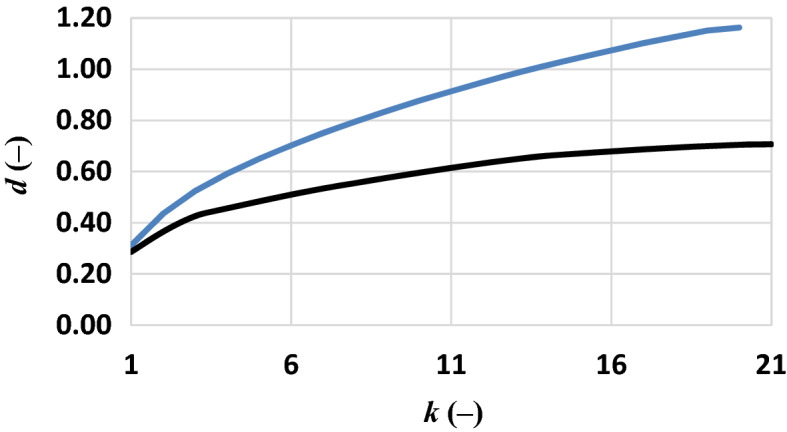
Spectral separability evaluated for the dry microplastics on this dataset (black line) and the literature dataset (blue line).

Several minima and maxima were evident in the range from 620 to 2100 nm for all microplastics; some of these were common in two or more polymers. Wavelengths corresponding to these “singularities” were selected for the further spectral separability, *d* (–), analysis (Fig. 3) except for those in the atmospheric absorption windows due to the water vapor; indeed, there latter could be feasible wavelengths for polymers detection through a laboratory experiment only.

Additionally, during the selection of suitable wavelengths those between them closer than 20 nm were merged. It was evaluated the derivative of the spectral separability, *d*′ (–). The knock of the *d*′ curve (not shown) suggests a best band combination (920, 1152, 1215 and 1298 nm) ensuring a separability of 0.46. The separability analysis over the spectral signature acquired in the literature dataset followed the same criteria (the 20 wavelengths used for this analysis were different from the wavelengths identified in Fig. 2). Results (Fig. 3, blue line) showed that the curve knee identified five bands (923, 1220, 1280, 1655 and 2170 nm) ensuring a separability of about 0.70.

**Figure 4:**
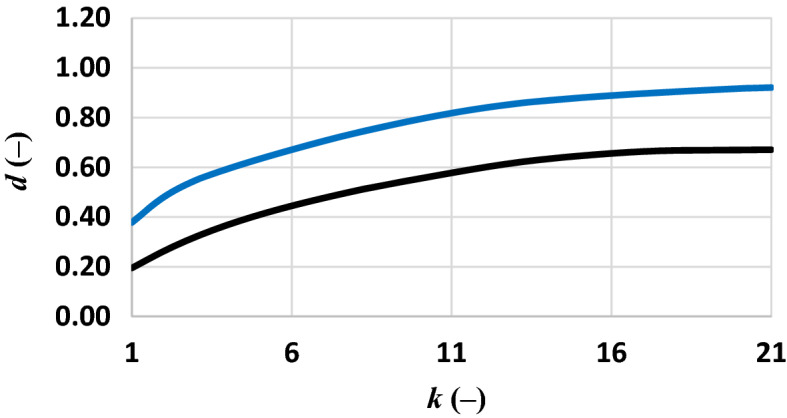
Separability analysis on PET, PS, PP of this dataset and PET, PS, PP of the literature dataset: best average separability of the dry microplastics (blue line, this dataset; black line, literature dataset) for given cluster of *k* bands.

The separability of common polymers (PET, PS and PP) was analysed for the dataset of the current experiment and that of the literature dataset. The wavelengths used for this analysis were those identified in the previous analysis (Fig. 2). The separability based on the dataset of the current experiment generated generally a higher value (Fig. 4).

### Microplastics on water

During the water-floating experiment, PET and PS were not tested as characterized by a density higher than that of the saltwater and, in the form of microplastics, they sank to the bottom of the experimental tank. Floating introduces the concept of object shape, since the flotation of an object does not depends only on the density of constituent material but also on its shape. As exemplificative, PET and PS constitute the main component of plastic bottles and cups, respectively. Thus, in a practical scenario, PET plastic bottles should be detectable since floating at the sea surface, while should not be detectable if fragmented in small pieces (plastic debris); while, PS small cups should not be detectable even in its commercial shape of plastic cups since these should possibly sink in the sea water.

**Figure 5:**
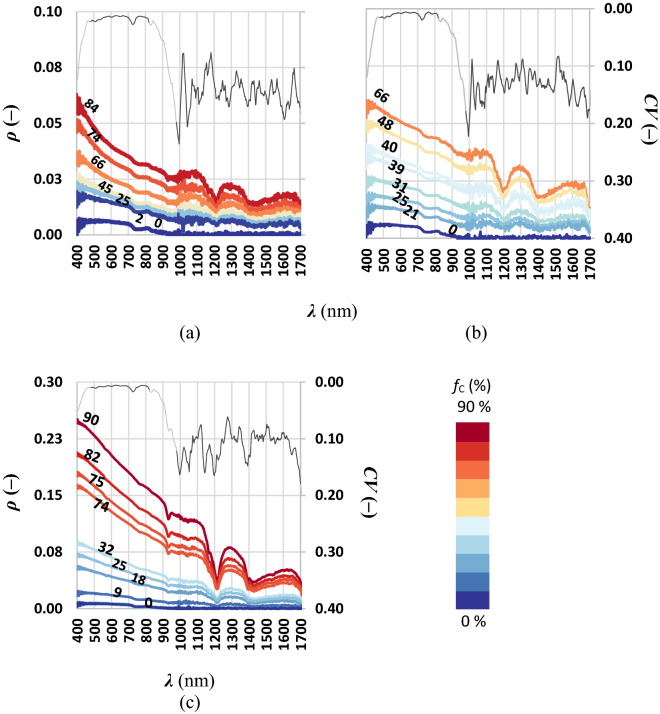
Spectral reflectances of water-floating microplastics: (**a**) EVA, (**b**) PP and (**c**) HDPE, for increasing *f*_C_. Light grey lines represent the *CV* averaged at 20 nm, while the *CV* values in selected spectral windows are represented in dark grey.

Spectral signatures on water floating samples with increasing of the areal fractional cover, *f*_C_ (%), was gradually brighter as expected. Noticeably, the water reflectance was noiseless from 480 to 820 nm (*CV *≈ 0.03) but noisy from 980 to 1700 nm (*CV *≈ 0.74). The average *CV* of the polymers reflectance (grey line, Fig. 5) was lower but showed a similar behaviour of that of water (the average *CV* was ≈ 0.01 between 480 and 820 nm for EVA, HDPE and PP, while it was ≈ 0.14, 0.11, 0.13 between 980 and 1700 nm respectively for EVA, HDPE and PP).

EVA and PP exhibited very low reflectance values (~ 6% at 400 nm with maximum of *f*_C_) while HDPE showed higher reflectance values (~ 25% at 400 nm with 90% of *f*_C_). The separability analysis was conducted considering four different pollution conditions.

**Figure 6:**
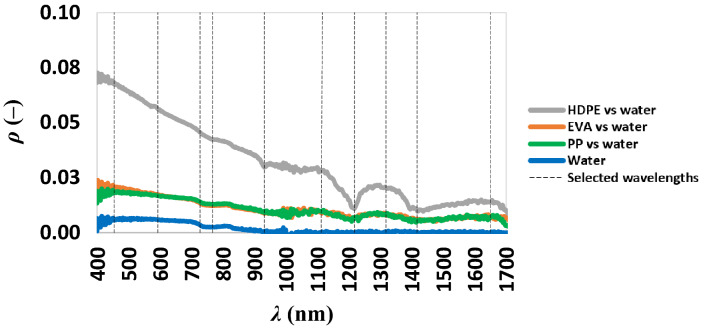
Spectral signatures of 25% for each microplastics on water and the spectral signature of water.

The fourth separability analysis was conducted considering the spectral signature corresponding to 25% of EVA, HDPE and PP and the spectral signature of water (Fig. 6), imposing that the analysis dealing with the case in which equal parts of three different polymers were floating in a water body (indeed, 25% of area is covered by water only).

This analysis allowed us to compare each microplastics polymer with water and each microplastics polymer with another one. Thus, these spectral signatures were compared and the suitable wavelengths were selected (Fig. 6) as before.

We considered the presence of the 50% for each polymer (EVA, HDPE, PP) floating alone in a water body. These analyses allowed to compare microplastics polymers with water showing low values of separability for each analysis. The separability of HDPE was higher than the other microplastics. The knee of the *d*′ curves (figure not shown) for HDPE and PP with water corresponded to the three band combinations (454, 593 and 727 nm). For the HDPE, such a band combination ensured a separability value equal to 0.159, while in contrast it reached a value of 0.072 for PP. The knee of the *d*′ curves for EVA with water corresponded to the four bands combination formed by the previous 3 bands with the addition of 1120 nm; this band combination ensures a separability of 0.03. The knee of the *d*′ curves, considering all together the four polymers, indicated a five bands combination (454, 593, 727, 767 and 931 nm) ensuring a separability of 0.052.

### Detection feasibility with current satellite sensors

The spectral separability analyzed on dry microplastics was compared with that evaluated by analyzing the spectral signature acquired in the literature dataset*.* The value of separability of all polymers in the literature dataset was higher than that measured in this experiment, probably because of the highest number of spectral bands in the first one (Table 1).

**Table 1:** Spectral separability of the dry microplastics in this experiment versus those of the literature dataset.

	Dry microplastics	
	This experiment	Literature dataset
*C* (*n,k*) (nm)	920–1152–1215–1298	923–1220–1280–1552–1655–2170
*d* (–)	0.46	0.702

Selected bands were similar in the two experiments although polymers were only partly coincident. Spectral signatures of the microplastics floating on water were considerably smaller than that on dry microplastics (this experiment). Indeed, water, having a high absorbance in the VNIR and SWIR strongly attenuates spectral reflectance in these wavelengths (Table 2).

**Table 2:** Separability of dry versus floating on water microplastics.

	Dry microplastics	Microplastics on water
*C* (*n,k*) (nm)	920–1152–1215–1298	454–593–727–767–931
*d* (–)	0.46	0.052

Additionally, the noise of the spectral signature of the microplastics on the water was greater than the dry microplastics and it was significantly higher for the wavelength greater than 900–1000 nm. Thus, the wavelength of the NIR and SWIR are less suitable to monitor microplastics on seawater. Results of the separability analysis based on the spectral signatures of the microplastics in water were compared with the bands of some currently existing sensors. The Ocean and Land Colour Imager (OLCI) sensor on board of Sentinel 3, has two bands of the five found in the range over which the spectral separability can be evaluated in this experiment. The bands reported in Table 3 could be used for the monitoring of microplastics on seawater; for this reason, it was conducted a specific separability analysis using the bands 767.5 and 931 nm.

**Table 3:** Bands candidates from OLCI (Sentinel-3).

OLCI (Sentinel-3)		
λ centre (nm)	767.5	940
Spectral resolution (nm)	2.5	20
Spatial resolution (m)	300	300

The separability with these two bands was equal to 0.025. This low value of the separability and the moderate spatial resolution of this sensor could make complicate the use of this sensor. Another useful sensor could be the WorldView-3. This sensor has four bands of the five found in the separability analysis of this experiment and the useful bands are reported in Table 4.

**Table 4:** Bands candidates from WorldView-3.

WorldView-3				
Bands (nm)	400–450	585–625	705–745	860–1040
Spectral resolution (nm)	1.24	1.24	1.24	1.24
Spatial resolution (m)	1.38	1.38	1.38	1.38

These bands could be used for the monitoring of microplastics on seawater; accordingly, the wavelengths 454, 593, 727 and 931 nm were used for the separability analysis. The separability with these four bands was equal to 0.048. The higher spatial resolution of the WorldView-3 than other sensors may increase the possibility to find high localized concentrations of microplastics.

Finally, the *PRecursore IperSpettrale della Missione Applicativa* (PRISMA) medium-resolution hyperspectral imaging mission by Italian Space Agency (ASI) was investigated. Main spectral characteristics of PRISMA are reported in Table 5.

**Table 5:** Bands useful for Prisma.

	Prisma	
	VNIR bands	SWIR bands
Spectral bands	66	171
Spectral range (nm)	400–1010	920–2505
Spectral resolution (nm)	≤ 12	≤ 12
Spatial resolution (m)	30	30

**Figure 7:**
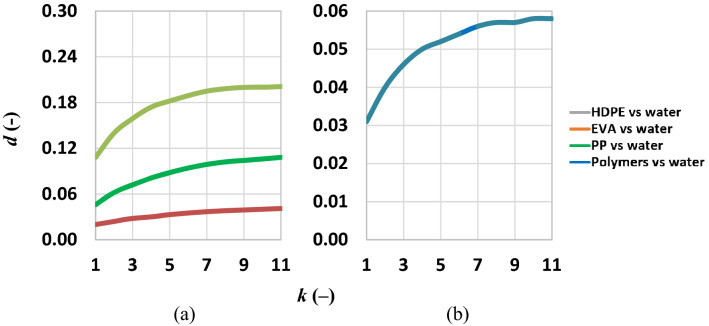
Lines represent the separability of microplastics from water at different *f*_C_: (**a**) separability is evaluated considering 50% of water and 50% of each polymer; (**b**) separability evaluated considering 25% HDPE, EVA, PP and water.

The spectral bands covering the wavelengths selected in Fig. 7 (panel b) are available in PRISMA.

Images are be acquired at 30 m spatial resolution. Using this sensor, an average spectral separability of about 0.058 is achievable.

## Discussion

The microplastics pollution at sea is a growing recognized problem. Microplastics pollution in our ocean, and in general in our environment, is increased in the last decades and it is foreseen that will reach 155–265 million tons of plastic accumulation by 2060^32^. In particular, the vortex of the oceans makes it a borderless problem; in fact, also countries that correctly allocate their waste, could have a plastics hotspot on their national seas, coming from other countries. The early detection—and the related early warning—of floating plastics hot spots across coastal areas may support the description of microplastic sources, pathways of arrival and spread at sea, as well as spatial–temporal trends of occurrences. An accurate and near real time detection will allow coastal socio-ecological systems to proactively react anticipating the introduction of microplastic on coastal biotic food webs and functioning. Near time detection brings clear benefits on socio-economies that rely on marine resources (e.g. fisheries-depending communities) and natural ecosystem services (e.g. human wellbeing and cultural heritage values associated to seascape and/or charismatic species^33^). Any technological improvement, as that presented here, may support societal multi-stakeholders’ efforts (i.e. from scientists, to environmental practitioners, policy makers and citizens) with the detection of microplastic at sea. It should be supported by research and innovation agendas (e.g. Horizon Europe 21–27) at international level. Satellite data, once validated, will provide a highly accurate and grounding knowledge for effective and efficient global understanding of microplastics associated risks and consequences, as already happening in various disciplines with other satellite-based data. In this context, any synergy among scientists and the most influential space agencies resulting into more suitable monitoring tool application and practice have been, and must be, encouraged and strengthened^34^.

Considering the difficulties encountered when monitoring microplastic pollution at sea through in situ surveys, here we proposed to evaluate the chance to use the remote sensing. The novelty of the experimental asset here performed is linked with the spectral characteristic analyses also of plastics in water. The approach allowed finding the best wavelengths (bands) generating the best separability for remote sensing monitoring.

Accordingly, the separability of the most common polymers found in the Mediterranean Sea was studied. The experiment was conducted on dry microplastics and water-floating microplastics. The presence of water can affect the use of the SWIR bands due to the absorption of water in this region of the spectrum.

The possible use of the current operational sensors was also investigated. The current sensor achieving the best average spectral separability (≈ 0.05 employing 4 bands at 1.38 spatial resolution) was the WorldView-3. Also, we showed that PRISMA sensor may be promising, since it seems to allow the best separability (≈ 0.06 employing 11 bands), even though at courser spatial resolution (30 m).

The future development of this work may include the acquisition of spectral signatures outdoor and the increase of the collection set of microplastics spectral signatures. Further, it will be necessary to consider other effects exerted on the sea surface by the atmosphere, the sunlight, the waves and the wind. Several studies have already shown that the microplastics are in suspension in the water column, so the sensor will have more difficult to identify the microplastics^35^ and also that we need to consider the spectral signature of the microorganism because the microplastics are a support of them, so they will cover the polymers.

## Methods

To identify suitable wavelengths and to distinguish different microplastic materials, a laboratory experiment was setup. The reflectance of each polymer was measured indoor using a spectroradiometer (see “Results”). Spectral signatures of polymers were collected over both dry and water-floating samples. Different acquisition strategies were designed for the dry and the water-floating experiments as explained in “Measurements on dry microplastics” and “Measurements on water-floating microplastics”.

The noise of spectral measurements was quantified through the coefficient *CV*, by rationing the standard deviation evaluated in a moving window of 11 nm to the mean.

The coefficient *CV* applied on the moving window highlights any spectral steep variation in the spectral reflectance besides the noise level. For this reason, the measurements noise is evaluated by quantifying the range of variability of *CV* over two wide spectral windows.

Thus, spectral separability *d* among the polymers signatures was evaluated for both the dry experiment and water-floating experiments. Separability was computed using the Euclidean formulation^36^:

1 $$d(x,\;y) = \sqrt {\mathop \sum \limits_{i = 1}^{n} (\rho_{{i_{j} }} - \rho_{{i_{t} }} )^{2} }$$

where:

- $\rho_{{{\text{i}}_{{\text{j}}} }} \;({-})$ and $\rho_{{{\text{i}}_{{\text{t}}} }} \;({-})$ represent the reflectances of the jth and tth spectral signatures, respectively;
- *n* (–) is the number of bands.

The higher *d* represents the better separability among spectral signatures. Separability was computed for given *n*, with a clustering of *k* (–) bands, thus leading to a possible number of band combinations, *C*(*n*, *k*), of:

2 $$C\left( {n,k} \right) = \frac{n!}{{\left( {k! \cdot \left( {n - k} \right)!} \right)}}$$

Separability among clusters is expected to be higher with increasing spectrally independent bands. Nonetheless, hyperspectral sensors are expensive and nowadays characterized by moderate/low spatial resolution and as a main consequence their large deployment is still less feasible for the detection of low area density microplastic. For this reason, *d*′ was computed to choose the set of bands, which allows achieving a relatively high *d* with the minimum number of *k* of bands. This approach could result advantageous when *d*′ tends to an asymptotic value with increasing set of band *k*. Thus, operationally, the best band combination has been chosen correspondently to the knee of the *d*′(*k*) curve.

### Measurements on dry microplastics

The reflectance on dry samples was measured by bounding polymers with a black ring placed on a black opaque fabric. Because the aim was to acquire the spectral signatures of the microplastic, the measures were carried out for increasing number of polymer layers, each one 0.18–0.20 cm in thickness, to avoid the influence of the underneath black panel of the emerging spectral radiance. Thus, for each polymer several spectral signatures were collected over the dry sample with increasing its total thickness. We chose as reliable polymer spectral signature that for which the reflectance differs less than 2% from the previous spectral signature (for each wavelength).

To optimize the computational efforts, only wavelengths corresponding to singularity points of the spectral signatures (*e.g.*, minima, maxima, knees, etc.) were used to compute the spectral separability.

The spectral signatures acquired in the present experimental work were compared with those reported in Garaba and Dierssen^37^, hereinafter referred as the literature dataset.

### Measurements on water-floating microplastics

In the present experiment, the microplastic samples were floating inside a black tank full of water. Tank walls were tick enough (~ 5 mm) to be considered characterized by negligible transmissivity. The tank was covered by a black opaque fabric to limit the effect on spectral measures of both the walls and the bottom of the tank. Spectral measures were carried out with different quantity of polymers with the aim to assess the effect of the fraction of polymers *f*_C_ seen by the optical fiber of the spectroradiometer. To compute the polymers areal fractional cover, a picture was collected contextually to each measure. Pictures were recorded and classified (using an unsupervised classification, namely a K-Means^38^ and cut according to the swath of the optical fiber of the spectroradiometer—constant as the acquisition geometry is fixed during the experiment). The fraction of area covered by polymers was obtained as ratio between the area classified as polymer and the swath area. Noticeable, for each polymer the surface covered by particles was different due to the dispersion of the particles on the water surface.

The separability was analysed by considering the presence of the 50% for each polymer (EVA, HDPE, PP) floating alone in a water body and the contextual presence of three microplastic’s types and water. To this aim, the interpolated spectral signatures corresponding to a *f*_C_ of 25% and 50% were used. The separability derivative allows identifying a knee assumed to represent the most suitable band combination. Indeed, although the separability rises with increasing bands’ combinations, the high costs of the sensors with many bands, or the low spatial resolution of hyperspectral sensor, could reduce the usefulness of these bands’ combinations.

### Types of plastic used

**Figure 8:**
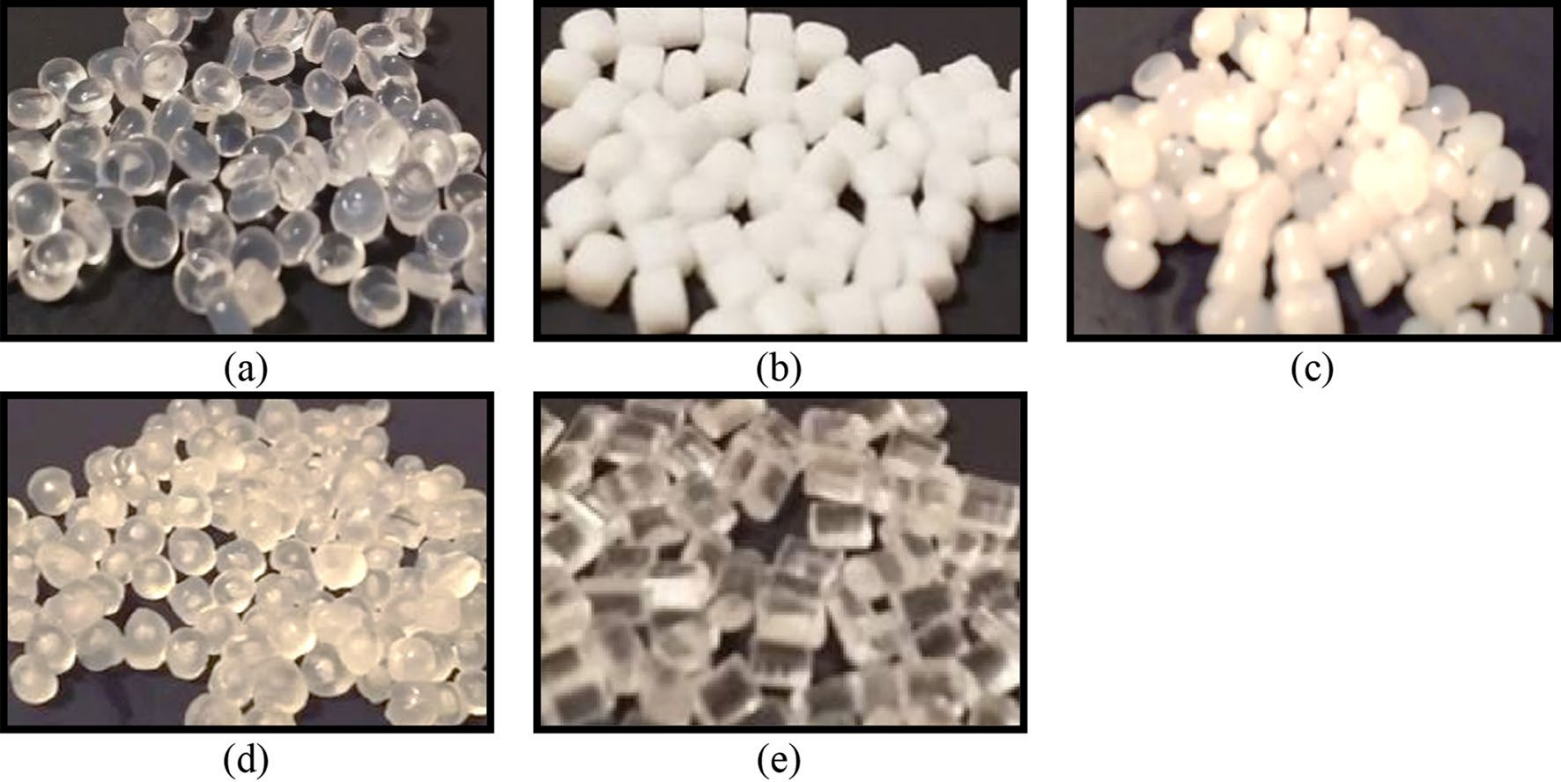
Spectrally characterized polymers: (**a**) ethylene vinyl acetate (EVA), (**b**) polyethylene terephthalate (PET), (**c**) high density polyethylene (HDPE), (**d**) polypropylene (PP) and (**e**) polystyrene (PS).

In this study microplastics among the most common in Mediterranean Sea were spectrally characterized and analysed (Fig. 8; EVA, HDPE, PET, PP and PS).

As already explained in “Microplastics on water”, during the water-floating experiments, PET and PS were not tested in the form of microplastics as they were not floating at the water surface. All the analysed microplastics were free of additives, thus almost transparent or white (Fig. 8). This is compliant with the real process, since most of the plastics lose the additives in the first phase of the degradation process.

### Experimental setup

**Figure 9:**
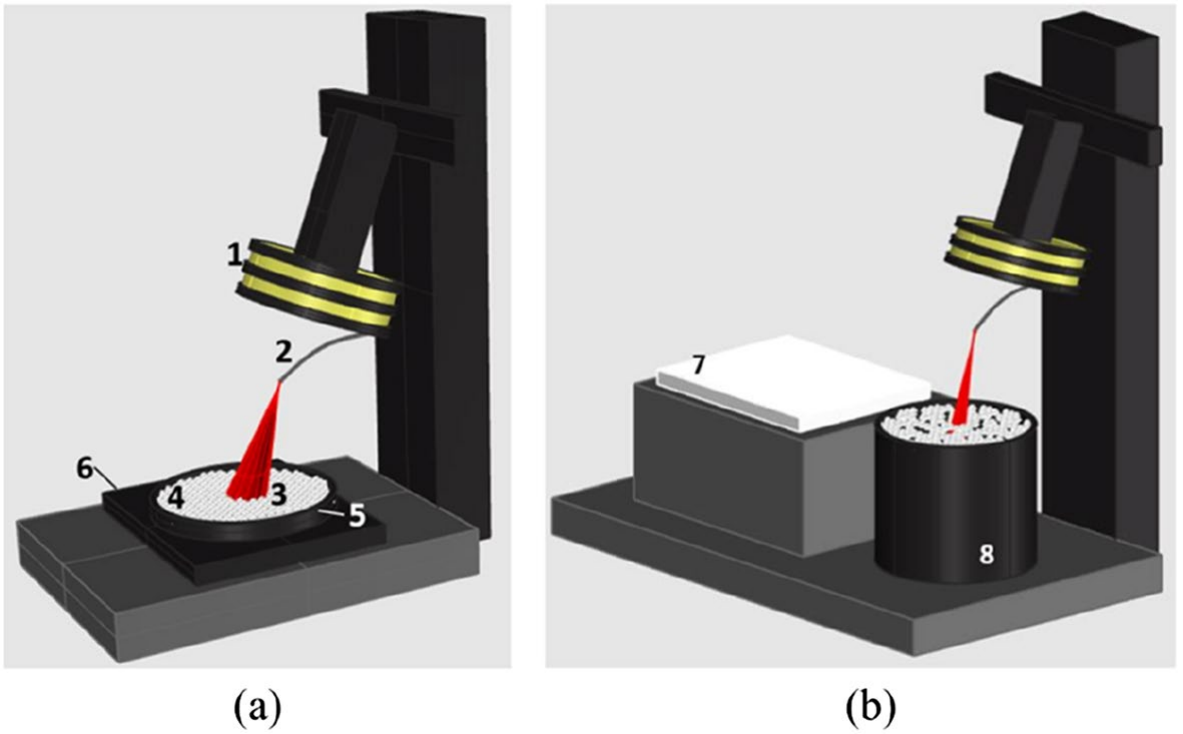
Spectroradiometric apparatus of (**a**) the dry microplastic and (**b**) the microplastics floating on water experiments. Indicated with numbers: (1) lamp, (2) optical fiber, (3) field of view, (4) microplastics, (5) ring, (6) black panel, (7) white reference panel, (8) tank.

Spectral reflectance was measured through a FieldSpec 4 Hi-Res spectroradiometer (by ASD, Analytical Spectral Devices) capable to cover the full solar reflected spectrum (from 350 to 2500 nm). The fiber optic had a field of view of 25°. During the measures on dry samples, a black panel of 16.2 × 16.2 cm (and 2 cm thickness) was used, as well as a black ring (9.2 cm in inner diameter; 1.8 cm height). The instrument wavelength accuracy is 0.5 nm, while Noise Equivalent Radiance characterizing the VNIR, SWIR 1 and SWIR 2 spectrometers are 1.0 × 10^–9^, 1.4 × 10^–9^ and 2.2 × 10^–9^ W cm^−2^ nm^−1^ sr^−1^ respectively at 700, 1400 and 2100 nm. During the experiment, a constant distance of 17 cm between the fibre optic and the upper surface of the polymer sample was imposed with varying layers thickness; the distance was kept constant by reducing the thickness of the samples and white panel base by unitary decrements of 0.1 mm to balance the increments of the sample thickness. Thus, minimizing the effect of the distance on the spectral irradiance reflected by the radiative source and incident the fiber optic. The radiative source area had thus a diameter of ≈ 3.8 cm (Fig. 9).

Operationally a white reference panel (in barium sulphate) was used to measure the incident irradiance. The black tank used to make the acquisition of the microplastics on water has a truncated cone shape. The inner upper diameter was 34 cm. It was coated with black opaque fabric, and then filled with saltwater to reach a depth of 27 cm. In this setup, the optic fibre was fixed at a distance of 27.5 cm from the water surface, thus the swath was ≈ 6.1 cm.

## Data Availability

All data generated during this study are available from the corresponding authors upon request.
